# Interactions between Obesity-Related Copy Number Variants and Dietary Behaviors in Childhood Obesity

**DOI:** 10.3390/nu7043054

**Published:** 2015-04-22

**Authors:** Dandan Zhang, Zhenli Li, Hao Wang, Min Yang, Li Liang, Junfen Fu, Chunling Wang, Jie Ling, Yan Zhang, Shuai Zhang, Yuyang Xu, Yimin Zhu, Maode Lai

**Affiliations:** 1 Department of Pathology, Zhejiang University School of Medicine, 866 Yu-hang-tang Road, Hangzhou 310058, Zhejiang, China; E-Mails: dandanz@zju.edu.cn (D.Z.); lizhenli8888@sina.com (Z.L.); brenda_1985@163.com (H.W.); ssnn@zju.edu.cn (S.Z.); 2 Key Laboratory of Disease Proteomics of Zhejiang Province, 866 Yu-hang-tang Road, Hangzhou 310058, Zhejiang, China; 3 Department of Nutrition, Zhejiang University School of Public Health, 866 Yu-hang-tang Road, Hangzhou 310058, Zhejiang, China; E-Mail: ymin36@zju.edu.cn; 4 Department of Pediatrics, the First Affiliated Hospital of College of Medicine, Zhejiang University, 79 Qing-chun Road, Hangzhou 310003, Zhejiang, China; E-Mails: zdliangli@163.com (L.L.); hzwangcl@yahoo.com.cn (C.W.); 5 Department of Endocrinology, Children’s Hospital of College of Medicine, Zhejiang University, 25 Guang-fu Road, Hangzhou 310003, Zhejiang, China; E-Mail: fjf68@yahoo.com.cn; 6 Department of Epidemiology & Biostatistics, Zhejiang University School of Public Health, 866 Yu-hang-tang Road, Hangzhou 310058, Zhejiang, China; E-Mails: lingjie_22@163.com (J.L.); joiny19@163.com (Y.Z.); jlt422519@live.cn (Y.X.)

**Keywords:** childhood obesity, risk, CNVs, dietary behaviors, interaction

## Abstract

Copy number variants (CNVs) have been implicated as an important genetic marker of obesity, and gene-environment interaction has been found to modulate risk of obesity. To evaluate the associations between CNVs and childhood obesity, as well as the interactions between CNVs and dietary behaviors, we recruited 534 obese children and 508 controls from six cities in China and six candidate CNVs were screened through published genome-wide studies (GWAS) on childhood obesity. We found three loci (10q11.22, 4q25 and 11q11) to be significantly associated with obesity after false discovery rate (FDR) correction (all the *p* ≤ 0.05). Cumulative effect of the three positive loci was measured by the genetic risk score (GRS), showing a significant relationship with the risk of obesity (*P*_trend_ < 0.001). The OR of obesity increased to 21.38 (95% CI = 21.19–21.55) among the 10q11.22 deletion carriers who had meat-based diets, indicating prominent multiplicative interaction (MI) between deletions of 10q11.22 and preference for a meat-based diet. Simultaneous deletions of 5q13.2 and duplications of 6q14.1 had significant MI with a preference for salty foods. Our results suggested that CNVs may contribute to the genetic susceptibility of childhood obesity, and the CNV-diet interactions modulate the risk of obesity.

## 1. Introduction

Obesity is a concerning public health issue within modern society, and childhood obesity has become a global epidemic [1]. The prevalence of childhood obesity has increased greatly in the past 25 years in China [2,3]. Childhood obesity is considered to be a risk factor for many chronic diseases such as hypertension [4], coronary artery disease [5], insulin resistance [6] and diabetes mellitus [7]. It is also associated with an increased risk of behavioral traits such as attention deficit hyperactivity disorder [8], lower intelligence [9] and various anxiety disorders [10].

Inheritance or genetic variations are crucial contributors to obesity, with high body mass index (BMI) heritability (0.47 to 0.90) estimated from twin studies [11]. Copy number variants (CNVs) are deletions or duplications of DNA segments ranging from one kilobase to several megabases, and are important constituents of genomic structural variants. A set of CNVs has been reported to be associated with early onset obesity by genome-wide analysis [12,13,14,15]. Deletions of 16p11.2 were first reported to be associated with severe early-onset obesity and developmental delay by Bochukova *et al.* [14]. Later, the results were replicated successfully by a larger case-control study utilizing array comparative hybridization [16]. A meta-analysis of 15 genome-wide analyses discovered that an upstream deletion polymorphism of NEGR1 (neuronal growth regulator 1) was a candidate variant of BMI [17]. Two subsequent genome-wide studies (GWAS) on childhood obesity detected the variant as well, which confirmed the importance of CNVs upstream of NEGR1 in childhood obesity [12,15]. Another important CNV is located at 10q11.22, which encompasses a BMI associated gene, PPYR1 (pancreatic polypeptide receptor 1), which was reported in a Chinese GWAS study [18]. Jarick *et al.* also detected copy number changes at 10q11.22 in European children [12].

Besides genetic factors, dietary behaviors play a vital role in obesity [19,20]. Recent studies suggested that genetic factors are involved in modulating the response to diet, thus affect susceptibility to obesity [21,22,23]. Hiroi M *et al.* reported that the PRDX3 gene was associated with obesity in subjects with high dietary fat intake, but not in subjects with low dietary fat intake [21]. An intervention study found that children with MC4R mutations had more difficulty regulating weight after lifestyle interventions compared to those without such mutations [24]. Such evidence provides a hint of gene-diet interactions in obesity, but the interactions between the main genetic factor—CNV—and diet have not been examined before.

The aims of the present study were to examine the associations of candidate obesity-related common CNVs in obese Han Chinese children and further explore the biological interactions of candidate CNVs and dietary behaviors.

## 2. Experimental Section

### 2.1. Subjects

The subjects were recruited from a cross-sectional study on metabolic syndrome of children and adolescents throughout six Chinese cities (Beijing, Shanghai, Tianjin, Hangzhou, Chongqing, and Nanjing) in 2010. A total of 1042 unrelated individuals (534 obese children and 508 non-obese samples) aged 7–17 were finally included in the study. Subject recruitment has been described in detail elsewhere [25]. The obese children were defined those with a BMI of greater than the 95th percentile in the same age and gender group, and individuals with a BMI between the 85th and 15th percentiles were defined as controls [26]. Samples were excluded according to the following criteria: (1) currently with or having a history of sever chronic disease or cancer; (2) possessing incomplete clinical information or a history of poor compliance; (3) having singular values in one or more areas of clinical or other study data; and (4) two or more individuals sharing a blood lineage (as evaluated via random selection). Obese cases and non-obese samples were frequency-matched by gender, age and living area. Informed parental consent of enrolled children was obtained for both blood collection and subsequent genetic analysis prior to the procedures. All experimental procedures were approved by the ethics committee of Children's Hospital, School of Medicine, Zhejiang University (Approval Number 2,009,020).

### 2.2. Anthropometric Measurements and Epidemiologic Investigation

Anthropometric measurements and epidemiologic investigation has been described previously [25]. In brief, anthropometric indices, including height, weight, waist circumference (WC) and hip circumference (HC) were measured. BMI, a person’s weight divided by their height squared, and waist to height ratio (WHtR), WC divided by height, were calculated. Data for dietary behaviors including dietary patterns (meat-based, vegetable-based or balanced diets), preference for salty foods (categorized as like, dislike or no strong preference) and preference for sweet foods (categorized as like, dislike or no strong preference) were collected via face-to-face interview by trained investigators using a standardized questionnaire.

### 2.3. CNVs Selection

Since rare variants are generally population specific and hard to replicate, we focused on the common CNVs in this study. We screened the published GWAS and aCGH (array-based comparative genomic hybridization studies) on childhood obesity compiled prior to December 2011; three GWASs and two aCGH studies were published [12,13,14,16,27]. However, only Jarick *et al.* [12] focused on the common variants. Therefore, common CNVs discussed in Jarick *et al.* were selected for this study [12]. Two CNV regions (11q11 and 1p31.1) suggesting significance in both discovery and replication datasets were included. In addition, we selected loci with adjusted *P* values of under 0.005 from case-control discovery samples. Thirteen loci were sent to be designed via Multiplex AccuCopy™ Kit (Genesky Biotehnologies Inc., Shanghai, China); they were 11q11, 1p31.1, 4q32.1, 3q21.3, 4q25, 5q13.2, 15q13.2, 10q11.22(1), 2p22.1, 10q11.22(2), 17q21.31, 1q21.1 and 6q14.1. Six loci were successfully designed for the CNV assay in one panel (see Table 1).

**Table 1:** Basic information of selected candidate CNV loci.

CNV_ID	Locus	Position *	Size (bp)	Overlap Genes
CNV_1	11q11	chr11: 55,130,596–55,210,165	79,569	OR4P1P OR4V1P OR4P4 OR4S2 OR4C6
CNV_2	5q13.2	chr5: 68,903,038–70,343,313	1,440,275	SMN1 SMN2 SERF1A SMA4 SMA5 GUSBP1 LOC643367 LOC643784 LOC653080 LOC653188 GTF2H2B LOC653391 LOC728488 SERF1B LOC728499 LOC728506 LOC728519 LOC728526 LOC728535 LOC728555 LOC100093625 LOC100132218 LOC100133280
CNV_4	10q11.22(1)	chr10: 46,338,178–46,812,351	474,173	GLUDP2 PPYR1 GPRIN2 SYT15 BMS1P2 LOC642826 LOC643650 ANXA8L1 CTGLF7 LOC728643 LOC728657 LOC100132646 FAM25B LOC100133189
CNV_10	10q11.22(2)	chr10: 47,011,183–47,145,122	133,939	LOC340844 LOC728684
CNV_12	6q14.1	chr6: 81,340,896–81,346,266	5370	None
CNV_13	4q25	chr4: 108,285,188–108,293,270	8082	None

* Chromosome positions were based on 2006 (NCBI36/hg18); CNV, copy number variation.

### 2.4. CNVs Genotyping by AccuCopy™ Technique

One and a half milliliters fasting whole blood samples were collected and frozen at −40 °C for subsequent DNA extraction. The TOYOBO MagExtractor Genomic DNA Purification Kit (Toyobo, Osaka, Japan) was used to extract DNA from whole blood. DNA concentration and purity were measured by Nano drop 2000 (Thermo Scientific) and DNA was stored at −80 °C. Qualified DNA was used to detect the copy number changes utilizing AccuCopy^®^ assay, a technology of multiple competitive real-time Polymerase Chain Reaction (PCR) developed by Genesky BioTech (Shanghai, China) [28]. In brief, the CNV detection assay for each sample was carried out in a 20 μL reaction containing 1 × Multiplex PCR Master Mix, 1 × Fluorescence Primer Mix, 1 × Competitive DNA mix, and ~10 ng genomic DNA. The primers for the target genes are listed in Supporting Information Table S1. The PCR program was as follows: 95 °C 10 min; 11 cycles of 94 °C 20 s, 65 °C–0.5 °C/cycle 40 s, 72 °C 1.5 min; 24 cycles of 94 °C 20 s, 59 °C 30 s, 72 °C 1.5 min; 60 min at 60 °C and keep at 4 °C to completion. PCR products were diluted 20-fold and loaded on ABI 3130XL sequencer (Life technologies, Carlsbad, CA, USA) for quantification detection.

### 2.5. Statistical Analysis

All statistical analyses were performed using SPSS version 19.0 (SPSS Inc., Chicago, IL, USA). Participants with missing CNV genotype data were excluded from the analysis. Normality of distribution of continuous variables was evaluated by Kolmogorov-Smirnov test. The quantitative data were described as means ± standard deviations (SD) for normally distributed variables, and median (lower quartile–upper quartile) for non-normally distributed data. Independent *t*-test for normally distributed variables, Mann-U Whitney test for non-normally distributed variables and χ^2^ test for categorical variables were utilized to examine the differences between obese and non-obese subjects. The standardized data of Chinese children was not available, so the WHO 2007 growth reference was used to calculate the age- and sex-specific BMI *z*-scores in order to exclude the effects of age and sex [29]. Odds ratio, 95% confidence interval and *P* values of deletions or duplications for the risk of obesity were computed via logistic regression adjusted for gender and age, using a normal CNV state (two copies) as reference. The same analysis was done when using the suggested age and gender specific cut-off of BMI by the International Obesity Task Force (IOTF) to assess obesity status [30]. The *p*-values were further adjusted by false discovery rate (FDR) using R software (version 2.15.3). Covariance analysis was used to evaluate the BMI *z*-score and WHtR difference between different copy number states of the positive loci using sex and age as covariates. For multiple comparisons, the least significant difference (LSD) test was used. Genetic risk score (GRS) was calculated as the number of the CNV risk alleles of the three positive loci on obesity, and chi-square trend test was performed to evaluate the dose response association between the GRS and the risk of obesity.

A multiplicative model of interaction was used to assess interactions between candidate CNV loci and dietary behaviors. As the effects ranging from deletions to duplications of the same loci were not necessarily linear, the interactions of different CNV states (deletion *versus* normal, duplication *vs.* normal) between dietary behaviors were calculated separately. Next, interaction terms were added to a multivariate logistic regression model to examine effects of MI, adjusted for sex and age. The bootstrap method with 1000 re-sampling was used to estimate the odds ratio and a 95% confidence interval of the logistic regression to reduce small sample bias. AP (attributable proportion due to interaction) and its 95% CI were calculated to evaluate the proportion of the risk of obesity due to interactions between candidate CNV loci and dietary behaviors [31].

## 3. Results

Five obese and ten non-obese children failed in the genotyping assay and thus were excluded in the analysis. The clinical characteristics of the remaining 529 obese children and 498 non-obese children were summarized in Table 2. The obese group had higher a BMI, BMI *z*-score, WHtR, WC, and HC compared with the control group (all *p* < 0.001). Approximately 23.5% of the obese children preferred a meat-based diet compared to 11.0% of the normal weight children (*p* <0.001). A significantly higher percentage of cases than controls preferred salty foods (25.8% *vs.* 10.8%, *p* < 0.001). No significant difference in preference for sweet food was observed. Subjects who preferred salty food and a meat-based diet had a significantly increased risk of obesity.

The association analysis results of the six CNV loci are shown in Table 3. Deletions of three candidate loci (11q11, 10q11.22 and 4q25) demonstrated significant difference in frequencies between cases and controls (*p* < 0.05). The significance persisted in all the three loci after multiple corrections by FDR. The deletion of 10q11.22 was closely associated with an increased risk of childhood obesity (OR = 2.40, 95% CI = 1.43–4.00; *p* = 9.1 × 10^−4^). Similar results were observed for 4q25 (deletion in 4q25: OR = 3.77, 95% CI = 1.52–9.36, *p* = 0.004). In addition, the three CNV loci showed consistent results when the obese definition of the IOTF was applied: the deletions of 11q11 and 10q11.2 were significantly associated with obesity after FDR correction (both *p* < 0.05) and 4q25 showed marginal significance, with a *p*-value of 0.06 (Supporting Information Table S2).

**Table 2:** Basic characteristics of the study subjects.

Characteristics	Obese Children (*n* = 529)	Non-Obese Children (*n* = 498)	*p*-Value
Age (years)	11.7 ± 2.9	11.7 ± 2.9	0.85
Gender (M/F)	345/184	326/172	0.93
BMI (Kg m^−2^)	27.3 ± 4.2	17.6 ± 2.3	<0.001
BMI *z*-score	2.71 ± 0.71	−0.17 ± 0.88	<0.001
WHtR	0.54 ± 0.06	0.41 ± 0.03	<0.001
WHR	0.89 ± 0.08	0.81 ± 0.06	<0.001
WC (cm)	82.8 ± 13.4	60.4 ± 8.1	<0.001
HC (cm)	96.0 (82.0–102.0)	75.0 (66.0–84.0)	<0.001
Food preference (*n*, %)			<0.001
Meat-based diet	120 (23.5%)	54 (11.0%)	
Balanced diet	358 (70.1%)	392 (79.7%)	
Vegetable-based diet	33 (6.5%)	46 (9.3%)	
Salt preference (*n*, %)			<0.001
Like	130 (25.8%)	53 (10.8%)	
No strong preference/dislike	374 (74.2%)	439 (89.2%)	
Sweet taste (*n*, %)			0.75
Like	319 (66.6%)	327 (67.6%)	
No strong preference/dislike	160 (33.4%)	157 (32.4%)	

HC was expressed as median (lower quartile–upper quartile), and other variables were expressed as means ± SD; *p*-values were calculated by independent *t*-test for normally distributed continuous variables; Mann-U Whitney test for non-normally distributed variables and χ^2^ test for categorical variables comparing obese and non-obese subjects; BMI, body mass index; BMI *z*-score, BMI for sex and age *z*-score; WHtR, waist to height ratio; WHR, waist to hip ratio; WC, waist circumference; HC, hip circumference.

Furthermore, the association analysis of the three positive loci showed consistent results on obesity-related index, BMI *z*-score and WHtR (Table 4). Deletions of 10q11.22 and 4q25 were significantly associated with higher BMI *z*-score and WHtR (all *p* < 0.01), and 11q11 showed marginal significance with the two indexes (*p* = 0.063 and *p* = 0.014 for BMI *z*-score and WHtR, respectively).

**Table 3:** Associations between candidate CNV loci and the risk of obesity.

Locus	Type	Obese Children (*n*, %)	Non-Obese Children (*n*, %)	OR (95%CI) ^1^	*p*-Value ^2^	*p* (FDR) ^3^
11q11	normal	218 (41.2)	232 (46.6)	1		
	deletion	123 (23.3)	92 (18.5)	1.43 (1.03–1.98)	0.03	0.05
	duplication	188 (35.5)	174 (34.9)	1.15 (0.87–1.52)	0.32	0.75
5q13.2	normal	386 (73.0)	369 (74.1)	1		
	deletion	68 (12.9)	59 (11.8)	1.10 (0.76–1.61)	0.61	0.61
	duplication	75 (14.2)	70 (14.1)	1.02 (0.72–1.46)	0.90	0.90
10q11.22	normal	459 (86.8)	464 (93.2)	1		
	deletion	52 (9.8)	22 (4.4)	2.40 (1.43–4.00)	9.1 × 10^−4^	0.006
	duplication	18 (3.4)	12 (2.4)	1.51 (0.72–3.18)	0.28	0.75
10q11.22(2)	normal	490 (92.6)	472 (94.8)	1		
	deletion	25 (4.7)	15 (3.0)	1.61 (0.84–3.10)	0.15	0.21
	duplication	14 (2.6)	11 (2.2)	1.23 (0.55–2.73)	0.62	0.87
6q14.1	normal	270 (51.0)	257 (51.6)	1		
	deletion	202 (38.2)	180 (36.1)	1.07 (0.82–1.39)	0.61	0.61
	duplication	56 (10.6)	61 (12.2)	0.87 (0.58–1.30)	0.50	0.87
4q25	normal	500 (94.5)	487 (97.8)	1		
	deletion	23 (4.3)	6 (1.2)	3.77 (1.52–9.36)	0.004	0.01
	duplication	6 (1.1)	5 (1.0)	1.16 (0.35–3.83)	0.81	0.90

^1,2^ Odds ratio, 95% confidence interval and *p*-values of deletions and duplications were calculated by logistic regression adjusted for gender and age, using normal CNV state as reference; ^3^ *p* (FDR) values represented the adjusted *p-*values for false discovery rate. CNV, copy number variation.

**Table 4:** Associations between the three positive CNV loci and BMI *z*-score, WHtR.

Locus	Type	BMI *z*-Score ^1^	*p*-Value ^3^	WHtR ^2^	*p*-Value ^4^
11q11	normal	1.25 ± 1.55		0.47 ± 0.08	
	deletion	1.49 ± 1.69	0.063	0.49 ± 0.09	0.014
	duplication	1.29 ± 1.72	0.649	0.48 ± 0.08	0.258
10q11.22	normal	1.26 ± 1.61		0.47 ± 0.08	
	deletion	1.87 ± 1.68	0.002	0.50 ± 0.08	0.006
	duplication	1.75 ± 2.12	0.069	0.51 ± 0.12	0.022
4q25	normal	1.29 ± 1.64		0.48 ± 0.08	
	deletion	2.18 ± 1.45	0.007	0.52 ± 0.09	0.008
	duplication	1.31 ± 1.74	0.852	0.51 ± 0.08	0.187

^1,2^ BMI *z*-score and WHtR of each CNV state was presented as means ± SD; ^3,4^ *p*-Values were calculated by covariance analysis with adjustment for gender and age, comparing the BMI *z*-score and WHtR of deletion or duplication group with the copy number = 2 group; WHtR, waist to height ratio; CNV, copy number variation; BMI, body mass index.

**Figure 1:**
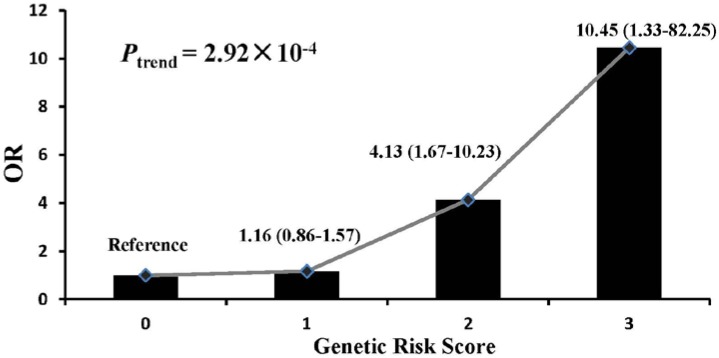
Cumulative effects of risk CNV loci on obesity. GRS (*X*-axis) was the sum of the CNV risk alleles of the three positive loci (11q11, 10q11.22, 4q25). Odds ratio (*Y*-axis) was calculated by logistic regression adjusted for gender and age, using the group with no risk type of the three loci as reference. The *P*_trend_ was calculated by Chi-square trend test.

To analyze the cumulative effect of associated CNVs, all subjects were divided into four groups according to the GRS (GRS = 0, 1, 2, 3). The ORs of each group were calculated using the group with GRS equal to 0 as reference (OR = 1). The ORs of the subjects with GRS equal to 1, 2 and 3 increased to 1.16 (95% CI = 0.86–1.57), 4.13 (95% CI = 1.67–10.23) and 10.45 (95% CI = 1.33–82.25), respectively. Significant dose-response relationship was observed between GRS and the risk of obesity (*p* for trend = 2.92 × 10^−4^) (Figure 1). The risk of obesity increased as the GRS increased.

The duplications of 6q14.1 showed a marginal association with preference for sweet foods. No significant association was observed between other CNV loci and dietary behaviors (Supporting Information Table S3).

**Figure 2:**
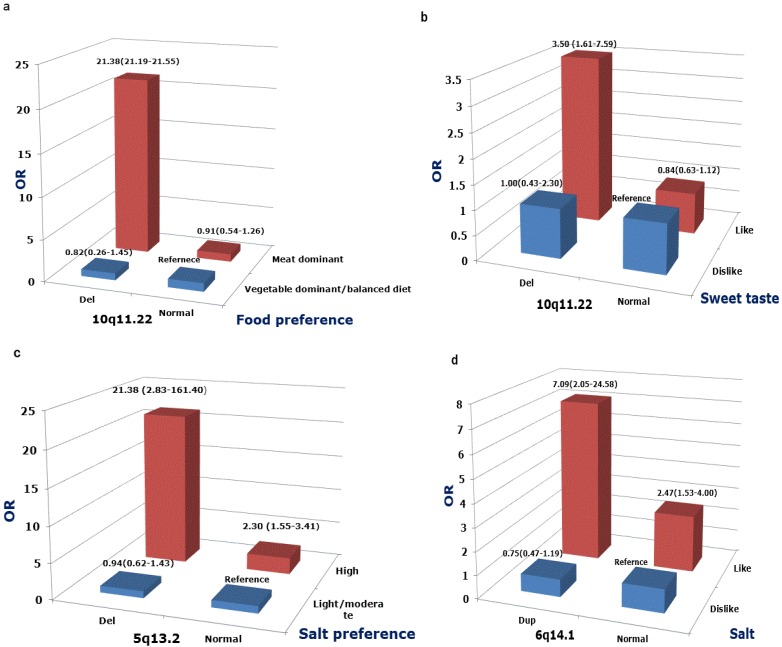
Positive CNV-diet interactions on the risk of obesity. Odds ratio (OR) (95% CI) for the risk of developing obesity were calculated by logistic regression adjusted for age and sex.

Interestingly, three CNV loci (10q11.22, 5q13.2, 6q13.1) showed significant MI with dietary behaviors (Details are shown in Supporting Information Table S4). Strong MI association between deletion of 10q11.22 and preference for meat-based diet was observed. All deleted carriers with meat-based preference had an increased risk of developing obesity, and the estimated risk (OR) for obesity was 21.38-fold (95% CI = 21.19–21.55) compared to reference (11q11.22 diploid case preferred vegetable-based or balanced diets) (Figure 2a). The OR of MI between deletions at 10q11.22 and preference for a meat-based diet was 19.64 (95% CI = 18.88–20.28), estimated by bootstrap.

Meanwhile, the deletion of 10q11.22 also revealed MI with sweet taste preference, with the OR of the interaction being 3.29 (95% CI = 1.10–9.83), and the AP being 0.76 (95% CI = 0.46–1.06) (Figure 2b). MI between 5q13.2 deletions and salty taste preference were also found to have an OR of 9.89 (95% CI =1.23–79.80), and the estimated AP was 0.90 (95% CI = 0.68–1.11) (Figure 2c). Similar results were also found between duplications of 6q14.1 and salty taste (OR for MI was 3.84, 95% CI = 0.97–15.28; AP = 0.69, 95% CI = 0.27–1.10) (Figure 2d).

## 4. Discussion

In the present study, we comprehensively evaluated 6 obesity-related CNV loci in childhood obesity of Han Chinese. We confirmed that CNVs at 10q11.22, 4q25 and 11q11 were associated with childhood obesity. Furthermore, significant interactions between 10q11.22, 5q13.2 and 6q14.1 and diet preferences were observed.

The deletions of 10q11.22 (CNV_4) demonstrated a highly significant association with the risk of childhood obesity in our study (*p* = 9.1 × 10^−4^). One study on Chinese population also detected that deletions at *PPYR1* were significantly associated with higher BMI [18], whereas another study found no copy number changes at *PPYR1* in 536 Chinese obese individuals and 263 healthy controls [32]. 10q11.22 contains 14 genes, and *PPYR1* is the most interesting gene in the region. *PPYR1*, also termed neuropeptide Y receptor Y4 (*NPY4R*), is one of the receptors of pancreatic polypeptide (PP). A series of studies have found that PP was associated with reduced food intake and lowered body weight [33,34,35]. Furthermore, it has been confirmed by several studies that PP regulates the intake of food via *PPYR1*; *PPYR1* knockout mice had no response to PP injections [36,37,38].

Interestingly, the interaction between deletion of 10q11.22 and dietary behaviors had prominent effects on the risk of obesity, especially the interaction with a preference for meat-based diets. The calculated AP for dietary pattern was almost 1, meaning that approximately 100% of the risk was due to the interaction. Although this might, to some extent, be due to the limited sample size of our study, it indicated strong interactivity between dietary patterns and 10q11.22 in obesity. Such strong interactions might be due to the regulation PP production. It has been well studied that protein and dietary fat can stimulate PP secretion, and glucose can also lead to a minor increase in PP levels [39]. Regularly, the intake of meat may stimulate PP secretion and PP regulating the intake of food via *PPYR1*. However, PP cannot function normally in subjects with 10q11.22 deletions. Thus, the 10q11.22 deletion carriers with meat-based diets might be more susceptible to developing obesity due to pathologic regulation of food intake. Interestingly, the 10q11.22 deletion carriers with meat dominant diets in the present study were all obese cases. In addition, the deletion of 10q11.22 also had significant multiplicative interaction (MI) with preference for sweet foods. This may be partly due to PP release stimulated by glucose consumption. These results may help explain how genetic factors responded to diet and are intricately involved in the pathophysiologic development of obesity, *i.e.*, how gene-diet interactions operate on a cellular level.

4q25 is a gene desert region, and deletions of CNV 4q25 conferred a highest risk of childhood obesity in our analysis (OR = 3.77, 95% CI = 1.52–9.36, *p* = 0.004). Little is known about the biological function of this region. There may reside some long-range regulators such as enhancers or repressors. CNVs may exert their effects on the causative gene that the distance can be as far as 1 Mb away [40]. Additionally, a marginal MI was shown between the duplication of 6q14.1 and salty preference. The duplication of 6q14.1 demonstrated no effect on obesity and the risk of obesity increased 7.09 (95% CI = 2.05–24.58) times when interacted with salty preference. Interestingly, Jarick *et al.* found that the duplication of the region was associated with obesity, whereas little is known about the region itself [12]. These data suggest that duplications at 6q14.1 play a role in the development of children obesity.

Not surprisingly, a strong cumulative association between risk alleles and childhood obesity was observed, with the risk of obesity increasing as individuals harbor greater numbers of CNV alleles at the three positive CNV loci (10q11.22, 4q25 and 11q11). The cumulative effect is highly significant with a *p*-value for trend of 1.98 × 10^−5^. We estimate that children with three risk alleles have an odds ratio of 10.45 for developing obesity. It may be possible to use the GRS to assess the risk of childhood obesity, but this strategy will have to be tested in a prospective study before being utilized for any such risk estimation.

## 5. Conclusions

In conclusion, our results provide support for the role of CNVs in the risk of childhood obesity, which is modified by lifestyle factors. The present study successfully replicated three obesity-related loci 10q11.22, 4q25 and 11q11 in Han Chinese children. A strong cumulative effect of these loci on the risk of obesity was noted. Furthermore, we observed strong MI between CNVs and dietary behaviors. Our findings highlight the importance of the interplay between genotypes and lifestyle factors. The full elucidation of complex interactions between genes and lifestyle factors may help establish public guidelines for the general population and have great implications on public health systems worldwide.

## Supplementary Files

Supplementary File 1
