# Supplementary material for: Interactions between Obesity-Related Copy Number Variants and Dietary Behaviors in Childhood Obesity
Source: Nutrients. 2015 Apr 22;7(4):3054–66. doi: 10.3390/nu7043054 (PMC4425189; doi:10.3390/nu7043054)
Supplement: Supplementary File 1 [file nutrients-07-03054-s001.docx]

**Supplementary Information**

**Table S1.** Primer sequences for target CNV regions in AccuCopy assay.

| **CNV_ID** | **Forward Primers (5'–3')** | **Reverse Primers (5'–3')** | **Position ^1^** | **Size (bp)** |
| --- | --- | --- | --- | --- |
| CNV_1 | TTCTATCACGTGCACCCAGCTC | TTTGGGGGTCACTGTGGATGT | chr11: 55,162,544–55,162,643 | 100 bp |
| CNV_2 | TCAATCCCACCACCCAAAATG | AGCTGGGGCAGGATATTGAGTT | chr5: 70,343,098–70,343,188 | 91 bp |
| CNV_4 | GTCCAGATAGGCTGGCAAGAGC | TCAGCTCAAGCTGCAAGGCTTA | chr10: 46,508,025–46,508,164 | 140 bp |
| CNV_6 | GCTACTGGCCCTGAAAGCAGGT | TGATCCAGCTCTTCCGTGAGAC | chr1: 72,560,431–72,560,580 | 150 bp |
| CNV_10 | CTGGGTCAGCATCCTCATCCTC | AATCTCTGTGGGGCCCAGTAAAA | chr10: 47,129,012–47,129,176 | 165 bp |
| CNV_12 | TGCGGAAGAAAAGAGGTGTTGT | AACCCCAGTGGAGCATTTCAGT | chr6: 81,345,804–81,345,881 | 78 bp |
| CNV_13 | CATCCTAGGGGTCATCACACACA | TCCATGAGGGACTATTTGGCACT | chr4: 108,291,424–108,291,557 | 134 bp |

^1^ Chromosome positions were based on 2006 (NCBI36/hg18).

**Table S2.** Associations between candidate CNV loci and the risk of obesity defined by IOTF

| **Locus** | **Type** | **Obese Children** | **Non-Obese Children** | **OR (95%CI) ^1^** | ***p*-Value ^2^** | ***P* (FDR) ^3^** |
| --- | --- | --- | --- | --- | --- | --- |
|  |  | **(*n*, %)** | **(*n*, %)** |  |  |  |
| 11q11 | normal | 134 (38.0) | 316 (46.9) | 1 |  |  |
|  | deletion | 90 (25.5) | 125 (18.5) | 1.43 (1.03–1.98) | **0.004** | **0.02** |
|  | duplication | 129 (36.5) | 233 (34.6) | 1.15 (0.87–1.52) | 0.08 | 0.16 |
| 5q13.2 | normal | 260 (73.7) | 495 (73.4) | 1 |  |  |
|  | deletion | 40 (11.3) | 87 (12.9) | 1.10 (0.76–1.61) | 0.51 | 0.612 |
|  | duplication | 53 (15.0) | 92 (13.6) | 1.02 (0.72–1.46) | 0.58 | 0.67 |
| 10q11.22 | normal | 303 (85.8) | 620 (92.0) | 1 |  |  |
|  | deletion | 35 (9.9) | 39 (5.8) | 2.40 (1.43–4.00) | **0.01** | **0.03** |
|  | duplication | 15 (4.2) | 15 (2.2) | 1.51 (0.72–3.18) | **0.04** | 0.12 |

**Table S2.** *Cont.*

| **Locus** | **Type** | **Obese Children** | **Non-Obese Children** | **OR (95%CI) ^1^** | ***p*-Value ^2^** | ***P* (FDR) ^3^** | |
| --- | --- | --- | --- | --- | --- | --- | --- |
|  |  | **(*n*, %)** | **(*n*, %)** |  |  |  |  |
| 10q11.22(2) | normal | 323 (91.5) | 639 (94.8) | 1 |  | |  |
|  | deletion | 16 (4.5) | 24 (3.6) | 1.61 (0.84–3.10) | 0.51 | | 0.612 |
|  | duplication | 14 (4.0) | 11 (1.6) | 1.23 (0.55–2.73) | **0.02** | | 0.12 |
| 6q14.1 | normal | 179 (50.9) | 348 (51.6) | 1 |  | |  |
|  | deletion | 130 (36.9) | 252 (37.4) | 1.07 (0.82–1.39) | 0.90 | | 0.9 |
|  | duplication | 43 (12.2) | 74 (11.0) | 0.87 (0.58–1.30) | 0.54 | | 0.67 |
| 4q25 | normal | 334 (94.6) | 653 (96.9) | 1 |  | |  |
|  | deletion | 16 (4.5) | 13 (1.9) | 3.77 (1.52–9.36) | **0.03** | | 0.06 |
|  | duplication | 3 (0.8) | 8 (1.2) | 1.16 (0.35–3.83) | 0.67 | | 0.67 |

^1,2^ Odds ratio, 95% confidence interval and *p*-values of deletions and duplications were calculated by logistic regression adjusted for gender and age, using normal CNV state as reference; ^3^ *P* (FDR) values represented the adjusted *p-*values for false discovery rate (FDR); IOTF: International Obesity Task Force.

**Table S3.** The association between candidate CNV loci and dietary behaviors adjusted for sex and age.

| **Locus** | **Type** | **Dietary Pattern ^1^** | ***p*-Value ^4^** | **Salt Preference ^2^** | ***p*-Value ^4^** | **Sweet Taste ^3^** | ***P* Value ^4^** |
| --- | --- | --- | --- | --- | --- | --- | --- |
| 11q11 | normal | 1 |  | 1 |  | 1 |  |
|  | deletion | 1.35 (0.88–2.08) | 0.17 | 1.27 (0.84–1.93) | 0.25 | 1.09 (0.76–1.57) | 0.65 |
|  | duplication | 1.20 (0.82–1.75) | 0.35 | 1.04 (0.72–1.51) | 0.83 | 0.89 (0.65–1.21) | 0.45 |
| 5q13.2 | normal | 1 |  | 1 |  | 1 |  |
|  | deletion | 1.05 (0.64–1.73) | 0.84 | 0.82 (0.48–1.39) | 0.46 | 0.93 (0.61–1.41) | 0.72 |
|  | duplication | 0.89 (0.54–1.47) | 0.65 | 1.13 (0.71–1.78) | 0.60 | 0.92 (0.63–1.36) | 0.69 |
| 10q11.22 | normal | 1 |  | 1 |  | 1 |  |
|  | deletion | 0.92 (0.48–1.77) | 0.81 | 1.10 (0.60–2.04) | 0.75 | 0.87 (0.51–1.47) | 0.60 |
|  | duplication | 1.43 (0.56–3.63) | 0.46 | 1.81 (0.78–4.19) | 0.17 | 1.18 (0.51–2.74) | 0.70 |
| 10q11.22(2) | normal | 1 |  | 1 |  | 1 |  |
|  | deletion | 1.46 (0.69–3.07) | 0.32 | 1.07 (0.48–2.38) | 0.87 | 0.72 (0.37–1.40) | 0.33 |
|  | duplication | 2.08 (0.84–5.17) | 0.11 | 1.93 (0.79–4.76) | 0.15 | 0.74 (0.32–1.73) | 0.49 |
| 6q14.1 | normal | 1 |  | 1 |  | 1 |  |
|  | deletion | 1.03 (0.73–1.47) | 0.86 | 1.09 (0.77–1.54) | 0.63 | 0.90 (0.67–1.20) | 0.46 |
|  | duplication | 0.84 (0.47–1.51) | 0.57 | 1.03 (0.61–1.75) | 0.91 | 1.85 (1.13–3.04) | **0.01** |
| 4q25 | normal | 1 |  | 1 |  | 1 |  |
|  | deletion | 1.32 (0.55–3.16) | 0.54 | 1.39 (0.58–3.36) | 0.46 | 1.16 (0.51–2.62) | 0.73 |
|  | duplication | 2.12 (0.54–8.34) | 0.28 | 0.42 (0.05–3.34) | 0.41 | 2.11 (0.44–10.21) | 0.35 |

^1,2,3,4^ Odds ratio and 95% confidence, *p-*values were derived from logistic regression adjusted for age and sex. ^1^ Food preference: reference group: vegetable-based or balanced diet; risk group: meat-based diet; ^2^ Salty preference: reference group: dislike or no strong preference; risk group: like; ^3^ Sweet taste: reference group: dislike or no strong preference; risk group: like.

**Table S4.** Interaction between candidate CNV loci and dietary behaviors on the risk of obesity.

| **CNV Loci** | | **Dietary Pattern ^1^** | | | **Salt Preference ^2^** | | | **Sweet Taste ^3^** | | |
| --- | --- | --- | --- | --- | --- | --- | --- | --- | --- | --- |
|  |  | **Case (*n*, %)** | **Control (*n*, %)** | **OR (95% CI) ^4^** | **Case (*n*, %)** | **Control (*n*, %)** | **OR (95% CI) ^4^** | **Case (*n*, %)** | **Control (*n*, %)** | **OR (95% CI) ^4^** |
| 5q13.2-del | | | | | | | | | | |
| normal | − | 290 (66.1) | 319 (75.6) | 1 | 279 (64.3) | 318 (75.4) | 1 | 114 (28.0) | 116 (28.0) | 1 |
| del | − | 46 (10.5) | 54 (12.8) | 0.95 (0.62–1.45) | 47 (10.8) | 57 (13.5) | 0.94 (0.62–1.43) | 20 (4.9) | 19 (4.6) | 1.07 (0.54–2.11) |
| normal | + | 84 (19.1) | 45 (10.7) | 2.18 (1.46–3.26) | 90 (20.7) | 46 (10.9) | 2.30 (1.55–3.41) | 233(57.2) | 242 (58.5) | 0.95 (0.69–1.31) |
| del | + | 19 (4.3) | 4 (0.9) | 5.43 (1.82–16.19) | 18 (4.1) | 1(0.2) | 21.38 (2.83–161.40) | 40(9.8) | 37 (8.9) | 1.08 (0.64–1.81) |
| MI (95% CI) ^5^ | |  |  | 2.63 (0.78–8.86) |  |  | **9.89 (1.23–79.80)** |  |  | 0.96 (0.77–1.20) |
| AP (95% CI) ^6^ | |  |  | 0.61 (0.15–1.06) |  |  | **0.90 (0.68–1.11)** |  |  | 0.05 (–0.75–0.86) |
| 10q11.22-del | | | | | | | | | | |
| normal | − | 339 (68.6) | 407 (84.8) | 1 | 324 (66.5) | 411 (85.6) |  | 144 (31.2) | 141 (29.8) |  |
| del | − | 39 (7.9) | 21 (4.4) | 0.82 (0.26–1.47) ^#^ | 39 (8.0) | 18 (3.8) | 2.77 (1.55–4.94) | 12 (2.6) | 12 (2.5) | 1.00 (0.43–2.30) |
| normal | + | 104 (21.1) | 52 (10.8) | 0.91 (0.54–1.26) ^#^ | 113 (23.2) | 48 (10.0) | 3.08 (2.13–4.47) | 273 (59.1) | 311(65.8) | 0.84 (0.63–1.12) |
| del | + | 12 (2.4) | 0 (0) | 21.38 (21.19–21.55) ^#^ | 11 (2.3) | 3 (0.6) | 4.84 (1.34–17.52) | 33 (7.1) | 9 (1.9) | 3.50 (1.61–7.59) |
| MI (95% CI) ^d^ | |  |  | **19.64 (18.88–20.28) ^#^** |  |  | 0.57 (0.13–2.40) |  |  | **3.29 (1.10–9.83)** |
| AP (95% CI) ^7^ | |  |  | **1.00 (1.00–1.00) ^#^** |  |  | 0.00 (–1.34–1.34) |  |  | **0.76 (0.46–1.06)** |

**Table S4.** *Cont.*

| 6q14.1-dup | | | | | | | | | | |
| --- | --- | --- | --- | --- | --- | --- | --- | --- | --- | --- |
| normal | − | 202 (64.1) | 225(71.9) | 1 | 195 (62.7) | 225 (71.9) |  | 86 (29.0) | 79 (25.6) |  |
| dup | − | 40 (12.7) | 57(18.2) | 0.78 (0.50–1.22) | 36 (11.6) | 56 (17.9) | 0.75 (0.47–1.19) | 10 (3.4) | 14 (4.5) | 0.66 (0.28–1.58) |
| normal | + | 59 (18.7) | 29 (9.3) | 2.28 (1.40–3.71) | 62 (19.9) | 29 (9.3) | 2.47 (1.53–4.00) | 159 (53.5) | 172 (55.7) | 0.86 (0.59–1.25) |
| dup | + | 14 (4.4) | 2 (0.6) | 7.81 (1.75–34.90) | 18 (5.8) | 3 (1.0) | 7.09 (2.05–24.58) | 42 (14.1) | 44 (14.2) | 0.88 (0.52–1.49) |
| MI (95% CI) ^d^ | |  |  | 0.78 (0.50–1.22) |  |  | ***3.84 (0.97–15.28)*** |  |  | 0.91 (0.64–1.29) |
| AP (95% CI) ^e^ | |  |  | 0.74 (0.32–1.15) |  |  | **0.69 (0.27–1.10)** |  |  | 0.42 (−0.35–1.19) |

^1^ Dietary pattern: +: meat-based diet; −: vegetable-based or balanced diet; ^2^ Salty preference: +: like; −: dislike or no strong preference; ^3^ Sweet taste: +: like; −: dislike or no strong preference; ^4^ Odds ratio, 95% confidence interval were calculated by logistic regression adjusted for age and sex; ^5^ MI (95% CI) represents the odds ratio and 95% confidence interval of multiplicative interactions calculated by logistic regression with adjustment for age and sex; ^6^ AP(95% CI）represents the attributable proportion due to interaction; ^7^ Odds ratio and 95% confidence interval were calculated using bootstrap method, based on 1000 bootstrap sample.

© 2015 by the authors; licensee MDPI, Basel, Switzerland. This article is an open access article distributed under the terms and conditions of the Creative Commons Attribution license (http://creativecommons.org/licenses/by/4.0/).
